# Biplanar *versus* conventional two-dimensional ultrasound guidance for radial artery catheterisation

**DOI:** 10.1016/j.bjao.2022.100122

**Published:** 2023-01-24

**Authors:** Harm J. Scholten, Gwen Broens, Michael I. Meesters, Joris van Houte, Renee J.C. van den Broek, Leontien ter Horst, Danihel van Neerven, Marjolein Hoefeijzers, Veerle Piot, Leon J. Montenij, Erik H.M. Korsten, R. Arthur Bouwman

**Affiliations:** 1 Department of Anaesthesiology, Catharina Hospital Eindhoven, Eindhoven, The Netherlands; 2 Department of Electrical Engineering, Eindhoven University of Technology, Eindhoven, The Netherlands; 3 Department of Anaesthesiology, Maastricht University Medical Centre, Maastricht, The Netherlands

**Keywords:** biplanar ultrasound, cardiothoracic anaesthesia, handheld ultrasound, radial artery catheterisation, ultrasound-guided vascular access

## Abstract

**Background:**

Ultrasound guidance increases first-pass success rates and decreases the number of cannulation attempts and complications during radial artery catheterisation but it is debatable whether short-, long-, or oblique-axis imaging is superior for obtaining access. Three-dimensional (3D) biplanar ultrasound combines both short- and long-axis views with their respective benefits. This study aimed to determine whether biplanar imaging would improve the accuracy of radial artery catheterisation compared with conventional 2D imaging.

**Methods:**

This before-and-after trial included adult patients who required radial artery catheterisation for elective cardiothoracic surgery. The participating anaesthesiologists were experienced in 2D and biplanar ultrasound-guided vascular access. The primary endpoint was successful catheterisation in one skin break without withdrawals. Secondary endpoints were the numbers of punctures and withdrawals, scanning and procedure times, needle visibility, perceived mental effort of the operator, and posterior wall puncture or other mechanical complications.

**Results:**

From November 2021 until April 2022, 158 patients were included and analysed (2D=75, biplanar=83), with two failures to catheterise in each group. First-pass success without needle redirections was 58.7% in the 2D group and 60.2% in the biplanar group (difference=1.6%; 95% confidence interval [CI], –14.0%–17.1%; *P*=0.84), and first-pass success within one skin break was 77.3% in the 2D group *vs* 81.9% in the biplanar group (difference=4.6%; 95% CI, 8.1%–17.3%; *P*=0.473). None of the secondary endpoints differed significantly.

**Conclusions:**

Biplanar ultrasound guidance did not improve success rates nor other performance measures of radial artery catheterisation. The additional visual information acquired with biplanar imaging did not offer any benefit.

**Clinical trial registration:**

N9687 (Dutch Trial Register).

Ultrasound (US) guidance increases the first-pass success rate and reduces the number of attempts and complications during radial artery catheterisation.^1^, ^2^, ^3^, ^4^ However, controversy exists over which US approach is superior: long-axis or in-plane view, or short-axis or out-of-plane view.^5^, ^6^, ^7^, ^8^, ^9^ The greatest disadvantage of short axis needling is the unnoticed advancement of the needle tip beyond the imaging plane, as the shaft is easily mistaken for the tip. This unintended advancement may lead to puncture of the posterior wall of the vessel.^6^^,^^7^ Dynamic needle tip positioning seeks to overcome this disadvantage as the transducer is moved along with the needle tip, but this technique requires extensive experience.^4^^,^^8^ Long-axis scanning more easily discriminates the needle tip from the shaft, but it is more difficult to learn and provides a reduced overview of the region of interest compared with short-axis scanning.^10^ Furthermore, superficial in-plane guided procedures can also be hindered by the beam width artifact.^11^ To complicate matters even further, the oblique axis has been suggested to be superior to both long and short axis, although the first-pass success rates for the control groups in the relevant studies were only 60–70%.^12^^,^^13^

Three-dimensional (3D) biplanar US imaging has the theoretical benefit of combining both long- and short-axis viewing, allowing simultaneous visualisation of both the needle including the tip (long axis) and the surrounding environment (short axis) during needling (Fig. 1). Nevertheless, evidence for improved accuracy or efficacy of vascular access procedures is limited to case reports for nerve blocks or phantom studies.^14^ The success rate of catheterisation of the internal jugular vein was improved with a cardiac 3D US probe. However, the resolution of this probe is inappropriate for superficial procedures, which may explain the low success rate in the control group of only 50%.^15^ A new compact handheld US device with adequate resolution for superficial procedures has been enabled with biplanar imaging.^16^ In a similar study regarding first-pass success rate of the internal jugular vein, no differences were found between the outcomes of 2D and biplanar guidance.^17^

**Fig 1:**
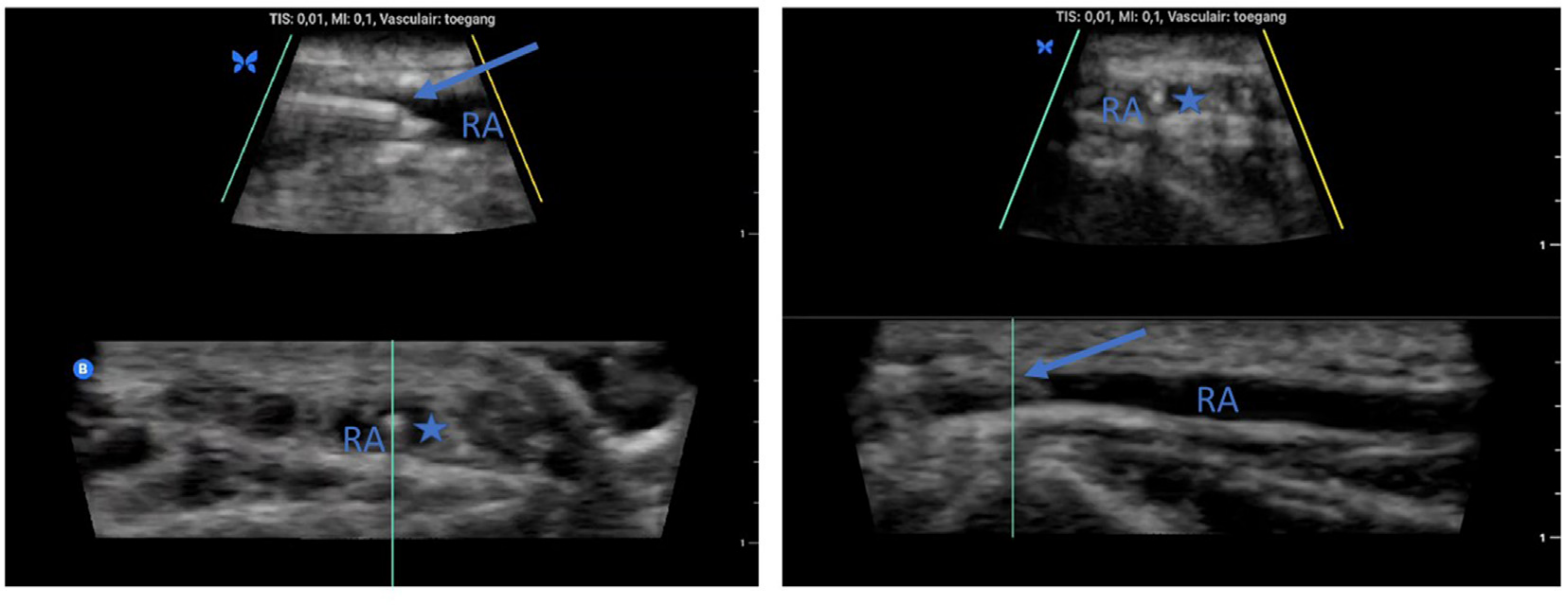
Biplanar catheterisation of radial artery. Left panel: upper image long-axis view, lower image short-axis view. Right panel: upper image short-axis view, lower image long-axis view. Arrow, needle; RA, radial artery; asterisk: needle tip.

As radial artery catheterisation is generally considered to be more difficult than internal jugular vein cannulation, we hypothesised that the enhanced anatomical awareness provided by 3D biplanar imaging could improve the accuracy of radial artery catheter placement. Hence, the aim of this study was to determine whether biplanar imaging provides superior guidance for radial artery catheterisation compared with conventional 2D imaging.

## Methods

This pragmatic, controlled, before-and-after clinical trial compared the outcomes of biplanar US guidance *vs* 2D US guidance for radial artery catheterisation. This single-centre study was performed at a large teaching hospital in the Netherlands. The trial was approved by the institutional review board MEC-U (NL78704.100.21; September 24, 2021), and written informed consent was obtained from all participants. Before patient enrolment, the trial was registered in the Dutch Trial register (NL9687). The trial was performed in agreement with the 1964 Declaration of Helsinki.

### Patient selection

The study included adult patients who required catheterisation of the radial artery for elective cardiothoracic surgery. Exclusion criteria were failure to obtain written informed consent, non-elective surgery, and anatomical abnormalities precluding arterial access through the radial artery (including abnormalities first noted on US imaging preceding the procedure). First, all eligible patients were included in the conventional 2D group. After completing the required number of 2D interventions, patients were admitted to the biplanar group for the remainder of the study. Blinding was not possible because of the nature of the intervention.

### Outcome data

**Fig 2:**
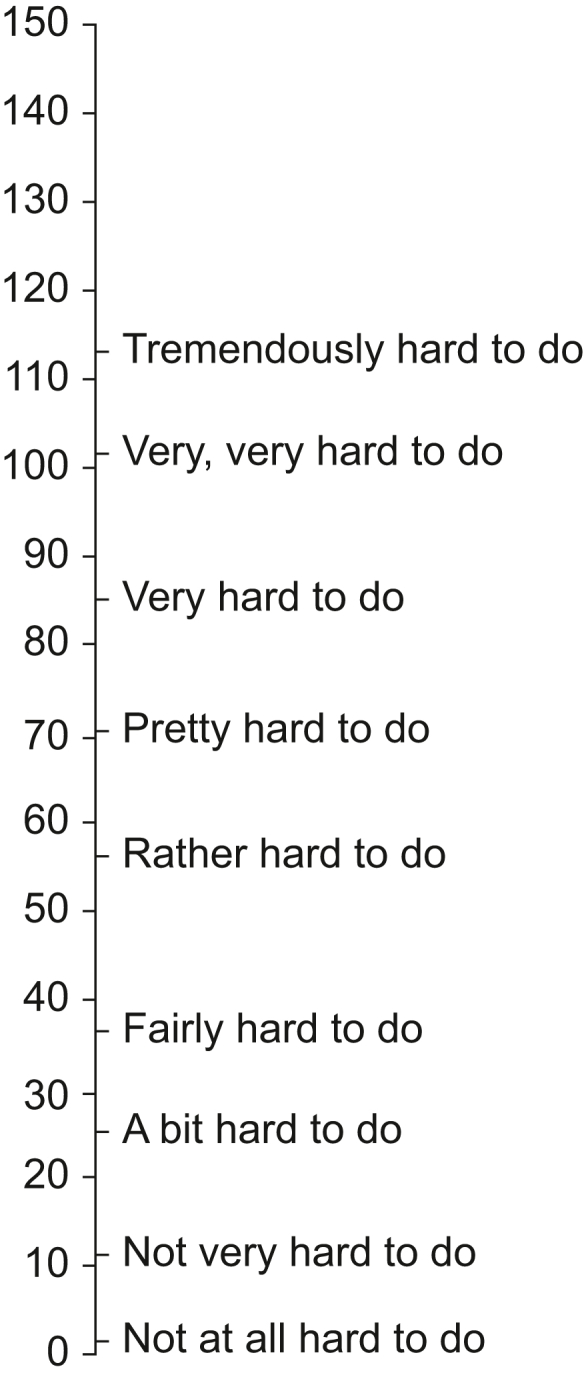
Subjective Mental Effort Questionnaire (SMEQ).^18^.

The primary outcome of the study was first pass success, defined as a successful radial artery catheterisation within one skin break and without needle redirections/withdrawals of more than 0.5 cm. Secondary endpoints were the total number of punctures and withdrawals needed for successful catheterisation, scanning time (from probe touching skin until skin break), needling time (from skin break until radial artery entrance and the appearance of blood in the chamber), and total procedure time (from probe touching skin until catheter placed in the radial artery), incidence of mechanical complications (haematoma, posterior wall puncture), needle visibility (good = needle visible, impression of the wall of the radial artery upon needle advancement and clear needle entry into artery; moderate = needle partly visible, or impression of the wall of the artery without visible needle; poor = no needle visible during radial artery entry) and operator-perceived mental effort (Subjective Mental Effort Questionnaire [SMEQ]).^18^ The SMEQ is a single-scale questionnaire measuring the mental effort required to perform a task. The scale ranges from ‘Not at all hard to do’ to ‘Tremendously hard to do’ (Fig. 2). All outcome data were recorded on a paper case record form by an observer.

### Study procedures

All participating anaesthesiologists were experienced in cardiothoracic anaesthesia and US-guided radial artery catheterisations, having performed more than 100 procedures. The majority of these individuals had already participated in a prior biplanar vascular access study.^17^ When required, they received instructions on biplane imaging and performed practice punctures in a phantom and at least 10 vascular access procedures before entering in the study. Both 2D and biplanar US scanning was performed with the Butterfly IQ+ ultrasound device (Butterfly Networks, Guilford, CT, USA).

In both groups, the patients' hands were positioned in dorsiflexion (approximately 45°) for optimal exposure of the radial artery. The entry site was anaesthetised with lidocaine 2% and the puncture was performed under aseptic conditions. Catheterisation was performed either with a Seldinger 20G radial artery catheterisation set with integral guidewire (Arrow International Inc./Teleflex Medical, Athlone, Ireland) or with a non-Seldinger 20G catheter with flow switch (BD, Franklin Lakes, NJ, USA), according to the anaesthesiologist's preference.

In the conventional 2D US group, the choice for long- or short-axis scanning was left to the discretion of the performing anaesthesiologist. Under real-time US guidance, the needle was advanced until the needle tip entered the artery, which was confirmed by blood entering the chamber of the needle. Either a guidewire was introduced through the needle and the catheter was advanced over the guidewire, or the needle was withdrawn with simultaneous advancement of the catheter, according to the type of catheter used. Intra-arterial placement was confirmed by the occurrence of an arterial waveform on the patient monitor.

In the biplanar group, the transducer was placed in transverse or longitudinal direction before the biplanar mode was selected (Fig. 1). The needle was inserted in a similar manner. The advancement of the needle was followed on the long-axis view, and the shadow and shaft of the needle were visualised on the short-axis screen, ideally at 12 o'clock in cross section. Entry of the radial artery, aspiration of blood, and guidewire confirmation were subsequently performed as described above.

### Sample size calculation and statistics

Based on previous RCTs,^4^^,^^8^^,^^19^ we expected first-pass success rates to be 80% in the 2D group and 95% in the biplanar group. Therefore, 75 subjects per group were needed to reach a power of 80% with an alpha of 0.05. To compensate for missing data, 10 additional patients were included, resulting in a total of 160 patients. All analyses were performed using SPSS (version 27.0; SPSS Inc., Chicago, IL, USA). Data were assessed for normality using the Kolmogorov–Smirnov test, and outcomes were presented as means (standard deviation [sd]) or median (IQR), as appropriate. With normally distributed data, continuous variables were analysed with Student's *t*-test and categorical variables with the χ^2^ test or Fisher's exact test. Non-normally distributed data were assessed with the Mann–Whitney *U*-test.

## Results

**Fig 3:**
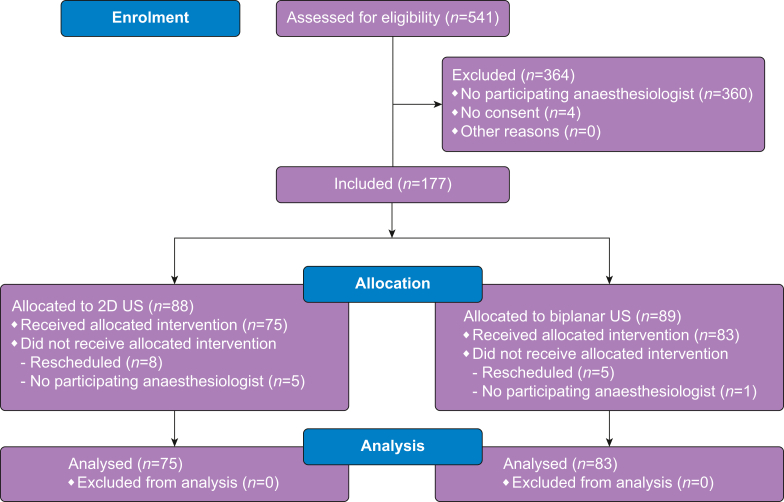
CONSORT flow diagram. CONSORT, Consolidated Standards of Reporting Trials.

Nine anaesthesiologists participated in this study. They performed from 10 to 32 procedures each. Between November 2021 and April 2022, 541 successive patients were assessed for eligibility. Of these, two-thirds were excluded as the anaesthesiologist was not participating in this study or the patient had already participated in another trial. Four patients refused to participate. Of the remaining 177 patients, 19 were excluded for logistical reasons (Fig. 3). In the final analysis, 158 patients were included. Baseline characteristics were similar in both groups except for the ASA classification (Table 1).

**Table 1:** Patient characteristics. Data are expressed as means with standard deviation. *P*-values for continuous data are based on Student's *t*-test for normally distributed data and Mann–Whitney *U*-test for non-normally distributed data. For % measures, the χ^2^ test was used. ADP, adenosine diphosphate; CABG, coronary artery bypass graft.

	2D	Biplanar	*P*
Age (yr)	68.8 (9.2)	68.8 (8.5)	0.584
Gender (male [%])	67 [90.7]	70 [84.3]	0.232
Weight (kg)	82.7 (15.2)	86.5 (15.3)	0.112
Height (cm)	174.1 (8.4)	175.8 (8.1)	0.204
BMI (kg m^−2^)	27.1 (3.9)	28.0 (4.9)	0.219
ASA classification			
3 (%)	21 (28.0)	41 (49.4)	0.006
4 (%)	54 (72.0)	42 (50.6)	
Peripheral vascular disease (%)	3 (4.0)	8 (9.6)	0.164
Anticoagulation (%)			
ASA	55 (73.3)	56 (67.5)	0.421
ADP inhibitor	26 (34.7)	33 (39.8)	0.509
Therapeutic	15 (20.0)	19 (22.9)	0.659
Procedure			
CABG	45 (60)	44 (53)	0.687
CABG + valve surgery	8 (10.7)	22 (26.5)	
Valve surgery	14 (18.7)	6 (9.2)	
Rhythm surgery	2 (2.7)	2 (2.4)	
Other	6 (8)	4 (4.8)	

Unsuccessful cannulations occurred twice in each group. The rate of first-pass success without redirections or withdrawals was similar for 2D (59%) *vs* biplanar (60%) guidance (*P*=0.84). Also, the rate of success within one skin break was similar (82% *vs* 77%, *P*=0.474). The required numbers of punctures and withdrawals, the imaging and procedure times, and the needle visibility did not differ. In the biplanar group, adequate visualisation of both short- and long-axis views was obtained in more than 90% of cases. Subjective mental effort was similar in both groups (*P*=0.186), with a mean (sd) score of 32 (26) for 3D guidance compared with 26 (26) for 2D guidance. All results are displayed in Table 2.

**Table 2:** Study outcomes. *P*-values for continuous data are based on the paired *t*-test for normally distributed data and on the Wilcoxon signed ranks test for non-normally distributed data. For % measures, the McNemar test was used. ∗Data are expressed as mean or as median (25th–75th percentiles).

Parameter	2D	Biplanar	Difference (95% CI)	*P*-value
First pass success (%)	58.7	60.2	1.6 (–14.0 to 17.1)	0.840
Within 1 puncture (%)	77.3	81.9	4.6 (–8.1 to 17.3)	0.473
Punctures (*n*)	1.09	1.11	–0.024 (–0.15 to 1.02)	0.886
Needle withdrawals (*n*)	0.72	0.66	0.058 (–0.29 to 0.41)	0.732
Posterior wall puncture (%)	8 (11.9)	17 (21.5)	–9.6 (–21.9 to 2.8)	0.126
Imaging time (s)∗	15.5 10 (7–20)	15.1 11 (8–16)	0.35 (–3.45 to 4.15)	0.349
Needling time (s)∗	38.8 24.5 (13.8–54.5)	35.5 29 (14–43)	3.2 (–7.85 to 14.3)	0.878
Procedure time (s)∗	58.8 44 (32.5–69.5)	56.8 44 (33–77)	1.98 (–10.75 to 14.70)	0.969
Needle visibility	2.45	2.31	–0.14 (–0.40 to 0.13)	0.304
Operator satisfaction	26.0	31.7	5.7 (–2.8 to 14.3)	0.186

Posterior wall puncture occurred relatively frequently, with 8 (12%) in the 2D group and 17 (22%) in the biplanar group, yet this difference was not statistically significant (*P*=0.126). The incidence of other mechanical complications was low, with just one local haematoma in each group. The choice of Seldinger (*n*=88, 56%) or non-Seldinger (*n*=70, 46%) technique did not lead to different first-pass success rates (78 *vs* 84%, *P*=0.580). Most anaesthesiologists preferred a transverse (short axis) probe orientation both during 2D (*n*=70, 93%) and biplanar (*n*=78, 94%). Transducer orientation did not influence first pass success rates (2D, *P*=0.65; 3D, *P*=0.58).

## Discussion

In this study, biplanar US guidance did not improve performance of radial artery catheterisation compared with conventional 2D US guidance, measured in terms of first-pass success rate. Furthermore, 2D or biplanar guidance did not result in significant differences in any of the secondary endpoints, including number of punctures, procedural times, and the anaesthesiologists' perceived mental effort.

Success at the first pass without redirections was approximately 60% in both groups, which is consistent with previous studies on improving radial artery catheterisation.^12^^,^^19^ Also, the observed success rates within one puncture of 73% and 80% are in line with the findings of earlier studies, although they are in the lower range of that spectrum.^6^ In particular, dynamic needle tip positioning has been reported to achieve successful cannulation within one skin puncture with fewer redirections.^4^^,^^20^ The slightly lower image quality and resolution of the compact handheld US device may be of greater importance relative to the small size of the target vessel than in the previous biplanar internal jugular vein studies.^15^^,^^17^ Furthermore, in the present study, the distance between the skin and the artery was relatively small, with less space for adjusting the direction of the needle once it was visible on the image.

The incidence of posterior wall puncture, which may lead to unsuccessful punctures, vasospasm, or haematoma, was lower than reported in the literature. However, posterior wall puncture still occurred quite frequently, with 11.9% in the 2D group and 21.5% in the biplanar group.^8^^,^^21^ Although this difference was not statistically significant, the better view of the needle tip with biplanar imaging may have revealed more posterior wall punctures than 2D scanning. In the 2D short axis view, the needle tip and shaft are difficult to distinguish, which means that a posterior wall puncture can go unnoticed as the tip can unintentionally be advanced beyond the US plane, perforating the posterior wall.

Success rates with either Seldinger or non-Seldinger technique were similar, reflecting that familiarity with the equipment is important for the outcome of a procedure. Scanning times were comparable for 2D and biplanar imaging, and the perceived mental effort was also similar, with scores ranging from fairly hard to a bit hard to do. Even for experienced anaesthesiologists, US-guided radial artery catheterisation apparently is not the easiest procedure to perform. As biplanar imaging did not increase the mental workload, it would be interesting to investigate whether biplanar imaging could benefit novice US users in obtaining competency in vascular access procedures.

3D or biplanar US has been investigated in catheterisation of the internal jugular vein, with varying results.^15^^,^^17^ For radial artery catheterisation, one case described successful catheter placement using biplanar imaging but no RCTs have been published yet.^16^ Despite the fact that biplanar imaging was not superior to 2D, this trial shows that radial artery catheterisation can be performed with a compact handheld US device without significantly decreasing clinical outcomes compared with more expensive and bulky ‘traditional’ US equipment. The low price of the device and especially its portability make it a valuable tool in acute settings, such as an unexpected event in an operating theatre or emergency room, where a high-end machine is not immediately available.

### Strengths and limitations

This study reflects daily clinical practice as it was performed by well-trained anaesthesiologists in a well-defined study population. However, our study has several limitations. First, we performed a before-and-after trial instead of a RCT. Baseline characteristics were similar between both groups except for the ASA classification, which should not have led to any bias. Also, a before-and-after design generally favours the intervention, which in this case means that it is even more reasonable to conclude that 3D biplanar guidance has no added benefit in radial artery cannulation. Second, the transducer orientation for 2D imaging was left to the anaesthesiologist's preference. Performing all procedures with either a transverse or a longitudinal orientation could have affected the results. However, transducer orientation probably did not significantly influence the results in the biplanar group, because short-axis and long-axis views were obtained simultaneously in this group in both approaches. Moreover, this pragmatic trial reflects clinical practice with varying anaesthesiologists' experiences and preferences regarding US-guided vascular access.

## Conclusions

Biplanar US-guided radial artery catheterisation was not superior to 2D guidance regarding first-pass success nor any of the secondary endpoints, including procedure times and operator mental effort. Handheld US imaging is feasible for radial artery catheterisation. Future studies are warranted to determine whether biplanar imaging would improve performance of novice US users, or whether biplanar imaging with higher resolution devices would influence the accuracy of experienced US users.

## Authors’ contributions

Study concept: HS, EK, AB

Study design: HS, AB

Acquisition of informed consent: HS, GB

Data collection: GB, MM, JvH, LH, DN, MH, LM, AB

Statistical analysis: HS

Developed the OLED-enhanced probe: RvB

Drafting of the manuscript: HS

Revision of the manuscript for important intellectual content: GB, MM, JvH, JvH, LH, DN, MH, LM, EK, AB

All authors approved the final version of the manuscript and agreed to have full accountability for all aspects of the work.
